# A new function of photochromic spiropyran: an efficient photoinitiator for two-photon polymerization

**DOI:** 10.1038/s41377-023-01188-1

**Published:** 2023-06-06

**Authors:** Xuanhang Wang, Bin Xu, Wenjing Tian

**Affiliations:** State Key Laboratory of Supramolecular Structure and Materials, College of Chemistry, Jilin University, Changchun, China

**Keywords:** Polymers, Optical techniques

## Abstract

Photochromic spiropyran molecule shows a brand new function for serving as an efficient photoinitiator to activate two-photon photopolymerization, thus paving the way for developing a fast 3D printing technology.

## Abstract (web summary)

New function of photochromic spiropyran molecules for initiating two-photon photopolymerization via direct laser writing holds great potential in fast 3D printing technology.

## Graphical abstract

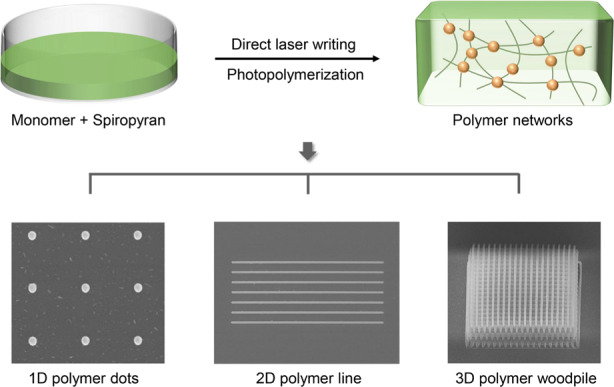

Recent booming development of two-photon photopolymerization that enables the conversion of liquid monomers into solid polymers by employing light as the energy shows great potential in advanced 3D printing technology^1^. The photoinitiator that acts as a key character in photopolymerization could generate reactive species of radicals or cations to initiate polymerization reaction once excited by the one-photon/two-photon absorption process^2^. Subsequently, photo-activated Norrish Type I or Norrish Type II photoinitiators will trigger the radical polymerization of acrylate monomers, one of the most widely used radical-initiated monomers, to realize the two-photon photopolymerization^3^. Unlike the direct formation of radical fragments of Norrish Type I photoinitiators under photoirradiation, Norrish Type II photoinitiators are two-component synergistic photoinitiating systems including sensitizers and co-initiators, commonly forming excited triplet state and requiring hydrogen donor to react^4^. The exploration of photoinitiators has been in progress to continuously optimize and improve the efficiency of two-photon photopolymerization.

Organic photochromic materials with controllable structural and optical switching properties play a critical role in advanced photonics, which mainly focus on the applications of optoelectronic devices, anti-counterfeiting display, optical memory, and super-resolution imaging^5–8^. Molecular photoswitches, an important class of photoactive chemical moieties, could reversibly alter their configurations between two isomeric states under different photoirradiation, thereby inducing absorption/luminescence changes and typical photochromic behaviors of materials^9^. Among the numerous photochromic materials, spiropyran (SP) molecules have raised much research interest because of their multifunctionality, which are not only in response to ultraviolet (UV) light irradiation but also sensitive to PH, solvent polarity and mechanical forces^10^. The isomerization of spiropyran molecules involves two continuous steps of molecular ring-opening and cis-trans isomerization process to change their structures from close orthogonal form to open merocyanine (MC) form^11^.

To date, research interests in spiropyran molecules mainly focus on the stimuli-responsive characteristics and optical switching capabilities, while their potential properties and functions in the field of photopolymerization are still unexplored. A recent publication in Light: Advanced Manufacturing by Baudrion et al. has provided a new discovery of photochromic spiropyran as a new photoinitiator to activate two-photon photopolymerization^12^. Researchers found that Nitro-substituted spiropyran could independently initiate the photopolymerization of pentaerythritol triacrylate (PETA) to fabricate 1D polymer dots, a 2D polymer line array, and even a 3D polymer woodpile (Fig. 1). Interestingly, the photopolymerization efficiency of PETA has gained significant promotion when adding a co-initiator into the formulation of spiropyran to provide hydrogen for photoreaction. Detailed experimental results have demonstrated that the introduction of the co-initiator does not quench the excited states of SP molecules and favors the formation of MC isomers.

**Fig. 1:**
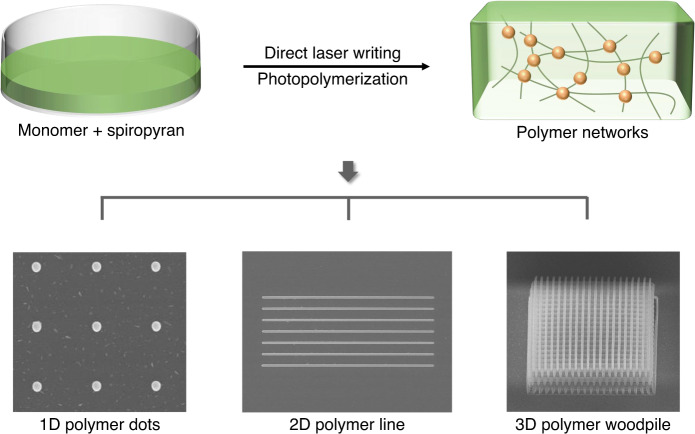
Schematic of photochromic spiropyran-initiated photopolymerization

Furthermore, the performances of different spiropyran derivatives in two-photon photopolymerization have been investigated in detail to reveal the intrinsic working mechanism. Normally, molecular isomerization of spiropyran derivatives could be induced by polar solvents even without UV light irradiation, thus forming two molecular configurations of apolar quinonic feature or dipolar zwitterionic character. This means isomeric components of spiropyran existed in the solution before the two-photon photopolymerization process. It’s worth noting that only the MC isomers with dipolar zwitterionic character could efficiently initiate the photopolymerization reactions. In contrast, the formulations containing indolino-8-methoxy-spirobenzopyran (8-MeO-BIPS) and indolinospiro-naphthopyran (NAP) that show apolar quinonic forms in polar medium, cannot initiate two-photon photopolymerization process. Given the evident correlation between photopolymerization efficiency and dipolar zwitterionic MC isomers, researchers considered radicals could arise, at least in part, from the photooxidation of MC isomers. This study offers a new sight for the functional exploration of spiropyran derivatives, broadening the application of photochromic materials in advanced 3D printing technology.
